# Challenges in ARDS Definition, Management, and Identification of Effective Personalized Therapies

**DOI:** 10.3390/jcm12041381

**Published:** 2023-02-09

**Authors:** Denise Battaglini, Brigitta Fazzini, Pedro Leme Silva, Fernanda Ferreira Cruz, Lorenzo Ball, Chiara Robba, Patricia R. M. Rocco, Paolo Pelosi

**Affiliations:** 1 Anesthesia and Intensive Care, San Martino Policlinico Hospital, IRCCS for Oncology and Neuroscience, 16132 Genoa, Italy; 2 Adult Critical Care Unit, Royal London Hospital, Barts Health NHS Trust, Whitechapel, London E1 1BB, UK; 3 Laboratory of Pulmonary Investigation, Carlos Chagas Filho Institute of Biophysics, Federal University of Rio de Janeiro, Rio de Janeiro 21941-901, Brazil; 4 Department of Surgical Sciences and Integrated Diagnostics, University of Genoa, 15145 Genoa, Italy

**Keywords:** ARDS, acute respiratory distress syndrome, mechanical ventilation, phenotypes, pharmacologic therapies

## Abstract

Over the last decade, the management of acute respiratory distress syndrome (ARDS) has made considerable progress both regarding supportive and pharmacologic therapies. Lung protective mechanical ventilation is the cornerstone of ARDS management. Current recommendations on mechanical ventilation in ARDS include the use of low tidal volume (V_T_) 4–6 mL/kg of predicted body weight, plateau pressure (P_PLAT_) < 30 cmH_2_O, and driving pressure (∆P) < 14 cmH_2_O. Moreover, positive end-expiratory pressure should be individualized. Recently, variables such as mechanical power and transpulmonary pressure seem promising for limiting ventilator-induced lung injury and optimizing ventilator settings. Rescue therapies such as recruitment maneuvers, vasodilators, prone positioning, extracorporeal membrane oxygenation, and extracorporeal carbon dioxide removal have been considered for patients with severe ARDS. Regarding pharmacotherapies, despite more than 50 years of research, no effective treatment has yet been found. However, the identification of ARDS sub-phenotypes has revealed that some pharmacologic therapies that have failed to provide benefits when considering all patients with ARDS can show beneficial effects when these patients were stratified into specific sub-populations; for example, those with hyperinflammation/hypoinflammation. The aim of this narrative review is to provide an overview on current advances in the management of ARDS from mechanical ventilation to pharmacological treatments, including personalized therapy.

## 1. Introduction

More than 50 years since it was first recognized, the incidence and mortality of acute respiratory distress syndrome (ARDS) remains high. More than 3 million people are diagnosed with ARDS each year. The incidence among critically ill patients in intensive care units (ICUs) is 10% and the mortality rate is 40% [1,2].

ARDS is a clinical syndrome, not a disease, and it is characterized by diffuse alveolar damage, inflammation, and edema causing acute respiratory failure with impaired gas exchange and oxygenation. An epidemiological study of a large population in 50 countries reported that ARDS was poorly recognized (delayed or missed diagnosis in about 40% of patients) [1,3]. Global awareness of ARDS is increasing, and it is recognized as a heterogeneous syndrome with direct and indirect causes, presenting with a wide range of clinical and pathologic characteristics [4]. The heterogeneity of the ARDS population has shifted clinicians’ focus to treatable traits, thus enabling better understanding and application of precision medicine. The aim of this narrative review is to evaluate the evolution of mechanical ventilation strategies and pharmacologic treatments for ARDS and discuss the challenges of finding effective therapies in the era of personalized medicine.

## 2. Evolution of the Definition of ARDS

The clinical presentation of ARDS in the critically ill was initially defined in 1967 with a report based on 12 cases describing clinical and pathologic features such as hypoxemia, noncardiogenic pulmonary edema, reduced compliance, increased work of breathing and the need for positive pressure ventilation in association with several diseases, including pneumonia and sepsis [5]. In 1994, an American–European Consensus Conference established the first consensus regarding specific diagnostic criteria for ARDS, which were updated in the Berlin Consensus Criteria in 2012 [6]. The so-called Berlin definition classified patients depending on their level of hypoxia into “mild” (partial pressure of oxygen (PaO_2_)/fraction of inspired oxygen (FiO_2_) between 200 and 300 mmHg with positive end-expiratory pressure (PEEP) or continuous positive airway pressure (CPAP) ≥ 5 cmH_2_O), “moderate” (PaO_2_/FiO_2_ between 100 and 200 mmHg with PEEP ≥ 5 cmH_2_O), and “severe” (PaO_2_/ FiO_2_ ≤ 100 mmHg with PEEP ≥ 5 cmH_2_O) ARDS. Although the Berlin definition has helped stratify patients in clinical trials, ARDS remains a unique heterogeneous syndrome with different underlying causes and clinical presentations. Patients clinically classified as “severe” ARDS (PaO_2_/FiO_2_ ≤ 100 mmHg with PEEP ≥ 5 cmH_2_O) do not always present the typical pathophysiologic hallmark of diffuse alveolar damage at autopsy [7]. Similarly, it has been observed that patients with acute hypoxemic respiratory failure treated with high-flow nasal oxygen had the same biomarkers as patients on invasive mechanical ventilation with ARDS [8]. Some studies investigated the Berlin criteria under spontaneous breathing during the early phase of the syndrome. At an early stage, Coudroy et al. [9] identified patients with ARDS in the absence of positive pressure ventilation using PaO_2_/FiO_2_ < 300 and bilateral pulmonary infiltrates. The clinical validity of the recent definition of ARDS is not known, and trials performed to date that have enrolled patients based on this definition have frequently failed to find effective pharmacologic therapies. In 2015, Villar et al. [10] proposed a new classification based on four categories for mechanically ventilated patients, finding that the degree of lung dysfunction is heterogeneous when using PaO_2_/FiO_2_ < 150 and PEEP of 10 cmH_2_O. At 24 h, mortality was significantly different among the four groups based on severity. However, Caironi et al. [11] pointed out that the degree of PEEP used can greatly affect PaO_2_/FiO_2_, thereby making assessment of the severity of ARDS misleading. The authors suggested continued use of the Berlin criteria at 5 cmH_2_O PEEP to better assess lung recruitability and edema with minimal risk of bias.

The criteria used to define ARDS have evolved over time, but they still need to evolve even further to ensure the definition is also applicable in low-income settings where resources, such as blood gas analysis, are not widely available. The Kigali definition by Riviello et al. [12] showed that 4% of hospital inpatients meet the modified criteria for ARDS without a requirement for PEEP; ratio of pulse-oximetric oxygen saturation to FiO_2_ (SpO_2_/FiO_2_) of 315 or less; and bilateral opacities on lung ultrasonography or chest radiography. Kwizera et al. [13] also support these findings. The future of ARDS in the era of precision medicine strives toward identifying treatable traits, thus focusing on ARDS etiology, physiology, and biomarkers [14].

## 3. Supportive Therapies

**Figure 1:**
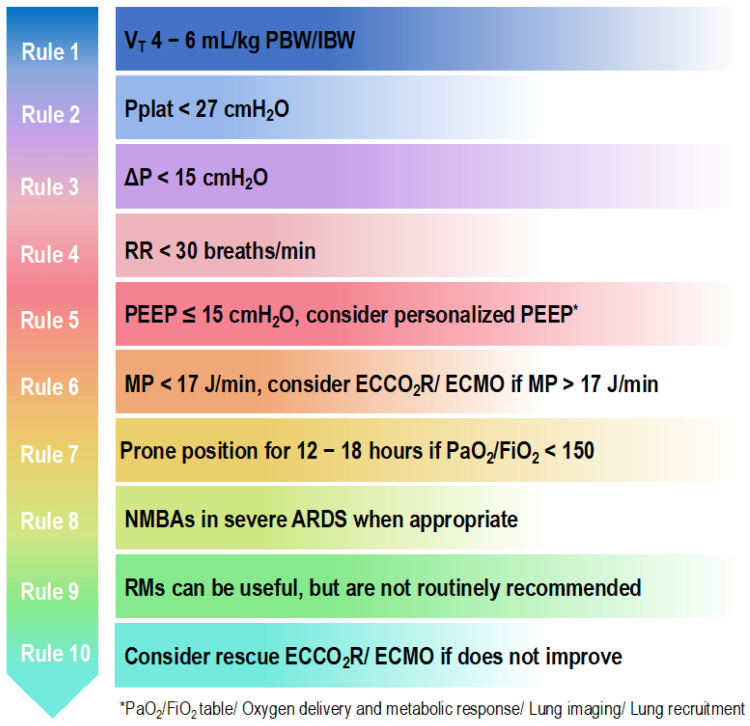
Ten golden rules to set the ventilator in patients with ARDS. V_T_, tidal volume; PBW, predicted body weight; IBW, ideal body weight; Pplat, plateau pressure; ∆P, driving pressure; RR, respiratory rate; PEEP, positive end-expiratory pressure; MP, mechanical power; ECCO_2_R, extracorporeal carbon dioxide removal; ECMO, extracorporeal membrane oxygenation; PaO_2_, arterial partial pressure of oxygen; FiO_2_, fraction of inspired oxygen; NMBAs, neuromuscular blocking agents; RMs, recruitment maneuvers.

Over the years, ARDS therapies have remained supportive, concentrating on the concept of protective mechanical ventilation strategies with the aim of mitigating ventilator-induced lung injury (VILI) [15]. Lung protective ventilation is standard practice, but the use of neuromuscular blocking agents and prone positioning are rescue strategies. We discuss these in detail. Suggested parameters of mechanical ventilation in patients with ARDS are shown in Figure 1.

### 3.1. Tidal Volume

The cornerstone of lung protective ventilation is the ARMA trial, which demonstrates that a low tidal volume (V_T_) equal to 6 mL/kg predicted body weight (PBW) compared with a higher V_T_ of 10–12 mL/kg PBW improved survival [16]. Recent trials and meta-analyses [17,18,19] recommend the use of low V_T_; however, low V_T_ ventilation is often underutilized for ARDS [20]. A multicenter international prospective observational study across 50 countries including 3022 patients with ARDS (LUNG SAFE trial) reported that ARDS was recognized only 60% of the time by clinicians, and less than two-thirds of patients with ARDS received a V_T_ ≤ 8 mL/kg PBW [1]. A study with 482 patients with ARDS found that every 1 mL/kg increase in V_T_ above 6.5 mL/kg was associated with a 23% increase in mortality in the ICU [21]. In addition, patients exposed to lower V_T_ (6 mL/kg PBW) from the beginning had an overall lower risk of ICU mortality compared with those who received higher V_T_ (8–10 mL/kg PBW) followed by lower V_T_ [21]. This is in contrast to the ARDSNet trials, which reported that ventilation with high V_T_ within the initial 48 h was not associated with increased hospital mortality [16].

Lung protective ventilation targeting low V_T_ and plateau pressure (Pplat) should start in the emergency department (ED). The LOV-ED investigators [22] found that the implementation of an ARDS protocol in the ED with low V_T_ and Pplat resulted in higher probability of using low V_T_ in the ICU, leading to less risk of VILI and lower mortality.

Current guidelines and clinical approaches suggest individualizing mechanical ventilation according to patient and disease characteristics targeting a V_T_ of 4–6 mL/kg without exceeding a V_T_ of 8 mL/kg PBW and target Pplat of ≤ 30 cmH_2_O [23] because higher values contribute to an overdistention of alveoli, leading to lung damage and mortality [24].

### 3.2. Positive End-Expiratory Pressure and Alveolar Recruitment

The application of PEEP results in benefits such as alveolar recruitment, reduction in intrapulmonary shunt, and increased arterial oxygenation [25]. Experimental studies demonstrated that PEEP could prevent possible lung injury from cyclic opening and closing and therefore protect patients from VILI by maintaining alveoli open that would otherwise become atelectatic or flooded at end-expiration (recruitment) [26]. However, PEEP also accounts for detrimental effects including increased end-inspiratory lung volume and increased risks of volutrauma and VILI by increasing lung stress and strain [27]. PEEP reduces cardiac output because a certain level of PEEP may increase pleural pressure and right atrial pressure, thus reducing venous return [28]. PEEP also increases pulmonary vascular resistance by narrowing or occluding alveolar septal vessels, thus increasing right ventricular afterload and further reducing cardiac output [28,29].

Four large randomized clinical trials (ART [30], ALVEOLI [31], ExPress [32], and LOV trials [33]) enrolling 3264 patients have compared higher PEEP (approximately 15 cmH_2_O) with lower PEEP (approximately 8 cmH_2_O or 13 cmH_2_O in the ART trial), and all failed to improve survival with higher PEEP, even if a trial suggested a survival benefit in sicker patients [34]. Interestingly, the ART trial reported higher mortality in the presence of high PEEP levels. This was not solely related to high PEEP levels; an extensive recruitment maneuvers (RMs) approach was also applied. Therefore, the underlying pathophysiology, lung mechanics, and degree of recruitability need to be monitored to evaluate the effects of PEEP. The explanation for these controversies may be associated with the application of PEEP in an unselected population, thus leading to overdistension and lung damage [35]. Moreover, high PEEP levels may result in detrimental hemodynamic effects, thus increasing shunt, dead space, and right ventricle afterload as well as reducing cardiac output [28,29]. The assumption that higher PEEP may lead to recruited lung units is not often observed, and it may result in overinflation and reduced lung compliance [36]. In the study by Constatin et al. [37] (the LIVE study), the authors adjusted the PEEP level as well as other strategies according to lung morphology. Patients in the control group received V_T_ of 6 mL/kg PBW and PEEP in accordance with a low PEEP and FiO_2_ table. Early prone positioning was encouraged. Patients with focal ARDS in the customized group underwent treatment with a V_T_ of 8 mL/kg, minimal PEEP and prone positioning according to the morphology of their lungs at imaging. Patients with non-focal ARDS received recruitment maneuvers, high PEEP, and a V_T_ of 6 mL/kg. This is an example of an attempt to personalize mechanical ventilation according to the pattern of disease.

If higher PEEP may be considered in a selected population, a meta-analysis of 18 randomized controlled trials including 4646 patients with moderate to severe ARDS showed that high PEEP with lung RMs may increase mortality [38]. This highlights the heterogeneity of individual patient response to PEEP strategies and the increasing interest in methods to personalize PEEP. To date, we remain unsure which is the best strategy to set PEEP and whether personalized PEEP strategies are a better option [30,39]. Recently, the concept of “keeping the lung at rest with permissive atelectasis and hypoxia” has been discussed. Oxygen consumption (VO_2_) is dependent on delivery (DO_2_), but meta-analyses did not confirm that implementing this parameter can offer advantages. However, it may be preferable to adopt a strategy accounting for cardiac output assessment, venous admixture levels (if ARDS is under normal metabolic response), carbon dioxide gap (PaCO_2_) as a marker of inadequate DO_2_, and PETCO_2_/PaCO_2_ to investigate those patients with ARDS who may need physiologic dead space monitoring as a global index of the efficiency of the lungs and PEEP setting [40,41].

### 3.3. Driving Pressure and Plateau Pressure

Driving pressure (∆P) or distending pressure represents the ratio between V_T_ and respiratory compliance, which can be easily obtained by Pplat−PEEP at the bedside [42]. ∆P offers an accurate picture of optimal lung mechanics in ARDS by estimating V_T_ and respiratory compliance, which correlates with aeration of the lung. Therefore, driving pressure represents an easy estimator of strain (V_T_/aeration of the lung at end-expiration) in ARDS.

The concept of “baby lung” in ARDS was defined as the fraction of lung parenchyma that still preserves normal inflation. Its size depends on the severity of ARDS and relates to static lung compliance. ∆P depends on the V_T_ as well as on the relative balance between the amount of aerated and/or overinflated lung at end-expiration and end-inspiration at different levels of PEEP [43].

In a retrospective analysis of 150 sedated and paralyzed patients with ARDS, Chiumello et al. [44] performed a PEEP trial from 5 to 15 cmH_2_O at a constant V_T_ and respiratory rate (RR), observing that at both PEEP levels, the group with higher driving pressure had significantly higher lung stress and lung elastance. Studies have reported the association between driving pressure and mortality in patients with ARDS and brain injury [45,46,47], suggesting the importance of using driving pressures as a strategy to target V_T_ and PEEP maintaining low stress. The LUNG SAFE study [48] showed that Pplat, PEEP, and ΔP were associated with ARDS prognosis and ΔP < 14 cmH_2_O was associated with decreased risk of hospital mortality in patients with moderate to severe ARDS. A recent study from Villar et al. [24] reported that Pplat was a more important determinant of mortality and outcome than ΔP. A meta-analysis of nine prospective trials involving more than 3500 patients showed that even when using lung protective ventilator settings (Pplat ≤ 30 cmH_2_O and V_T_ ≤ 7 mL/kg IBW), ΔP was the physical variable that best correlated with survival in patients with ARDS [45]. In conclusion, the literature suggests that ΔP should be kept below 13–15 cmH_2_O and used in association with low V_T_ and Pplat < 30 cmH_2_O as well as the lowest PEEP that can keep oxygenation at an acceptable value [49].

### 3.4. Slower Is Better

Lung tissue presents a viscoelastic behavior. It implies that stress is not constant during a sustained constant strain; for example, when the lungs are maintained inflated at a constant volume, the transpulmonary pressure decreases progressively with time. Tissue deformation can be expressed as strain, which is defined as the ratio of V_T_ over the end-expiratory lung volume for the lung. Strain has been used to determine safe thresholds of V_T_ to prevent VILI. In addition, the “strain rate” is the change in lung strain (deformation) with respect to time. Longer times are related to lower strain rates and shorter times are related to higher strain rates. This mechanism can be discussed within all the components of mechanical ventilation. Of utmost interest but never considered is the time at which the changes in ventilator setting are made [50]. As said before, ventilatory parameters do not always account for different time constants at both inspiration and expiration as well as inhomogeneity and heterogeneous ventilation of different alveolar units. Alveolar inflation and deflation manifest at different timings, even in a healthy lung; thus, at low inspiratory time constant, alveoli easily inflate, whereas at high inspiratory time constant, alveoli need more time to completely inflate. This reflects that at a high RR, even at a low time constant, alveoli will have less time to inflate. RR is often underconsidered, but recent findings have suggested its association with mortality and VILI [51]. Given the so-called stress relaxation of the lungs, parenchymal damage can be observed depending on how fast V_T_ or strain is modified for a period of time. Changes to V_T_ are often made abruptly, although the extracellular matrix requires a time of stress relaxation to mitigate the strain. It has been found that when the time of adaptation is shorter rather than abrupt, this attenuates lung injury. However, when the adaptation time is longer, this leads to more lung damage, suggesting that injurious strain is initiated in every case but can be decreased when using a shorter adaptation time [52]. RMs associated with improved oxygenation and lung mechanics have been identified as a potential cause of VILI. RMs need to exceed the critical opening pressure of the small airway to be effective [53], and alveoli recruit with a different time constant in heterogeneous lungs, thus requiring different timings to open each alveolar unit. This highlights the importance of rapid versus slow increases, given that sudden changes in airway pressure and flow increase stress and worsen lung damage [54]. Less VILI was observed in gradual versus abrupt increases in airway pressure [55]. In a recent meta-analysis, the use of stepwise increases in PEEP and/or RMs did not result in survival or less barotrauma compared with a strategy using a PEEP targeted at acceptable oxygenation goals [38]. However, the Survival Sepsis Campaign [56] does not indicate the use of stepwise RMs but instead advises abrupt increases given the results of the ART trial [30] and the PHEARLAP trial [57]. Focusing on PEEP, a recent study showed that lung damage can occur after sustained inflation followed by abrupt deflation, and this can be led by hemodynamic impairment followed by an increase in pulmonary microvascular pressure [58]. Similarly, Rocha et al. [59] investigated an abrupt versus gradual PEEP release combined with standard or high fluid volumes, finding that an abrupt reduction in PEEP, regardless of fluid status, causes greater epithelial cell damage and increases pulmonary arterial pressure. There are several other examples in the literature that may deserve further discussion. In conclusion, we need to highlight that slow changes in ventilatory parameters should be preferred over abrupt changes to limit further damage to the lung [60].

### 3.5. Mechanical Power

Mechanical power (MP) is the amount of energy transferred by the mechanical ventilator to the respiratory system per unit of time and is determined by the combined effects of applied V_T_, ∆P, RR, inspiratory flow, and PEEP, as well as determinants of mechanical properties of the lung (e.g., respiratory system elastance and airway resistance). MP might be a more accurate parameter for lung protective ventilation because it considers the balance of all individual ventilator parameters. MP is calculated using the following formulas: in volume-controlled ventilation, MP = 0.098 × V_T_ × RR × (Ppeak,RS − ΔP,RS/2); in pressure-controlled ventilation, MP = 0.098 × V_T_ × RR × (ΔP,RS + PEEP). An observational study found no causal relationship between the mechanical power and mortality, and MP normalized to the compliance or to the amount of well-aerated tissue was independently associated with ICU mortality [61]. In general critically ill patients, MP > 17 J/min was associated with higher mortality [62]. MP > 22 J/min was associated with increased 3-year mortality and 28-day mortality in patients with ARDS [63]. In a recent experimental study, MP > 25 J/min caused more significant and potentially lethal lung damage than lower values [64]. Similarly, in patients treated with extracorporeal membrane oxygenation (ECMO), MP > 14.4 J/min, during the first 3 days, was the only ventilatory variable independently associated with 90-day hospital mortality [65]. However, the benefit of MP is unclear, and its clinical use is limited by the complexity of measuring and interpreting it, and other variables, such as ∆P, are easily measurable at the bedside and are also predictive of mortality. Another interesting new parameter is the formula of Costa et al. [51] (4 × ∆P) + RR, which showed significant association with mortality (hazard ratio = 1.152, 95% confidence interval (CI) = 1.040–1.276, *p* = 0.006) and poor neurologic outcome (odds ratio = 1.244, 95% CI = 1.015–1.525, *p* = 0.036) with a better performance compared with MP in patients after cardiac arrest without ARDS. Despite its potential advantages, this formula has been tested only as an observational association with outcome and deserves further investigation. This formula has been studied recently in patients without ARDS post-cardiac arrest. In the secondary analysis from Robba et al. [66], the composite formula calculated as (4 × ΔP) + RR was independently associated with mortality and poor neurologic outcome. This shows that after cardiac arrest, ventilator settings (specifically ∆P and RR) in the first 3 days after hospital admission influence patient outcomes at 6 months.

### 3.6. Other Modes of Ventilation

Airway pressure release ventilation (APRV) is a ventilatory modality that uses continuous positive airway pressure and a partial and short release phase of ventilation that allows the patient to breath spontaneously [67,68]. The patient breaths spontaneously for a predetermined time with a high pressure of 20–30 cmH_2_O, decreasing to a low pressure according to the elastic recoil of the respiratory system, which is maintained with an expiratory flow around 25–50% of the maximum value [67]. The high and low pressures are usually set according to the P/V loop. In a recent meta-analysis, this novel ventilatory modality demonstrated good efficacy in improving oxygenation and shortening the ICU length of stay in patients with ARDS [68]. Zhong et al. [69,70] and Sun et al. [69,70] concluded that APRV increases compliance, oxygenation, and hemodynamics in comparison with a lung protective ventilator strategy. In addition, APRV resulted in reduced mortality, duration of mechanical ventilation, and ICU length of stay. Animal models suggest that the use of APRV over controlled mechanical ventilation reduces VILI when used with a V_T_ of 6–8 mL/kg PBW, optimal PEEP, and higher total amount of spontaneous breathing (30%–60% of total ventilation). Transpulmonary pressure seems to be lower in APRV than in controlled ventilation but using an RR equal to 50% of the controlled rate or pressure support equal to P high, avoiding P_0.1_ > 3–4 cmH_2_O [71,72,73]. Saddy et al. [71] found that different assisted ventilation modes led to improved lung function and reduced inflammation compared with pressure-controlled ventilation.

Similar to APRV, time-controlled adaptive ventilation during APRV improved lung recruitment and distribution of ventilation, thus reducing VILI, lung damage, and inflammation in an experimental model [74,75]. However, when setting APRV, we should always keep in mind that the data available are poor and that APRV represents a pressure control modality of ventilation with potential for volutrauma if not properly set [76]. Optimal targets of APRV come mainly from preclinical studies. However, during assisted mechanical ventilation, VILI can occur because of increased spontaneous breathing effort, patient–ventilator asynchrony, *pendelluft* and inhomogeneous ventilation, increased capillary perfusion due to alveolar edema, and tensile stress. During spontaneous inspiration, both tensile and compressive stress can occur. After inspiration, pleural pressure decreases, resulting in tensile stress on the extracellular matrix and an increase in capillary size but also compressive stress, which reduces capillary size. Evidence suggests that tensile stress is less harmful than compressive stress [77,78].

High-frequency oscillatory ventilation (HFOV) is another mode of ventilation that has been tested in patients with ARDS with contrasting findings. HFOV is actually not recommended by guidelines for its potential for high intrathoracic pressure, altered hemodynamic response on ventricular preload, pneumothorax, airway obstruction, acidosis, and cellular injury [79]. The potential rationale for its use is that HFOV can limit VILI, using V_T_ equal to or lower than dead space (up to 3 mL/kg), thus using high RRs (around 150 breaths/min) with a bias flow of 5–60 L/min [79]. Alveolar ventilation is determined by the following formula: (f) × (V_T_)^2^, thus maintaining a continuous distending pressure and facilitating the elimination of carbon dioxide. Despite proven reduced inflammation in an animal model of ARDS [80], HFOV in patients with ARDS improved oxygenation, but mortality was increased in those patients without severe hypoxemia [81,82,83,84]. This could be explained by the modest tidal volumes produced by HFOV, which are often equal to or lower than dead space. The lungs’ constant mean airway pressure and high respiratory rate both contribute to the maintenance of alveolar ventilation. Guidelines suggest using this modality only in cases of rescue therapy or as a research target in those patients who cannot tolerate high V_T_ and distending pressures.

### 3.7. Prone Positioning

Prone positioning in patients with ARDS improves oxygenation, increases recruitment potential, and reduces areas of alveolar overdistension, thus ensuring more homogeneous aeration of the lungs and potentially reducing VILI. The initial evidence from the PROSEVA trial was the first study to show a benefit of the prone position on mortality for patients with moderate to severe ARDS with PaO_2_/FiO_2_ < 150 mmHg with 60% FiO_2_ with PEEP of at least 5 cmH_2_O for at least 16 h until clinical improvement. In this study, prone position was associated with an improvement in mortality at day 28 (16% versus 33%, *p* < 0.0001), which persisted at day 90 (24% vs. 41%, *p* < 0·0001) [85]. As learned from COVID-19, the response to prone positioning depends on the redistribution of densities and regional perfusion [86].

International guidelines now recommend that the prone position should be instituted early and ideally within 36 h of meeting these criteria and should be used alongside lung protective ventilatory strategies [23,87].

The low incidence of prone positioning is partly explained by concerns regarding adverse events such as endotracheal tube obstruction, pressure sores, and loss of venous access [88]. However, prone positioning is also resource intensive and should be performed by a trained and experienced team. During ECMO, Giani et al. [89] recently showed that the prone position improved oxygenation, reduced intrapulmonary shunt, and reduced hospital mortality.

### 3.8. Extracorporeal Membrane Oxygenation and Extracorporeal Carbon Dioxide Removal

Venous–venous (VV)–ECMO has been considered as a rescue therapy for patients with severe ARDS due to the complications related to it and the controversial evidence. The multicenter CESAR trial [90] compared ECMO with conventional management of ARDS and showed that only 76% (n = 68/90) received ECMO, but this group had an improvement in the primary outcome of quality of life at 6 months. The subsequent EOLIA trial showed a signal toward improvement in mortality (relative risk, 0.76; 95% CI, 0.55–1.04, *p* = 0.09), but it did not achieve statistical significance, and a subsequent post hoc analysis showed that early ECMO was more beneficial. An individual patient data meta-analysis showed a statistically significant benefit in mortality at day 90 in the ECMO group (relative risk, 0.75; 95% CI, 0.6–0.94, *p* = 0.013) [90]. ECMO has also been shown to be effective in COVID-19, and a cohort of 7345 patients across five countries showed that ECMO was a deliverable therapy in 844 patients, and patients with a PaO_2_/FiO_2_ ratio <80 mmHg indicated ECMO was associated with reduced mortality compared with conventional therapy (relative risk, 0.78; 95% CI, 0.75–0.82) [91]. ECMO may have the ability to support lung protective ventilation and maintain low ΔP because a recent meta-analysis including more than 500 patients showed that ΔP during the first 3 days in ECMO had an independent association with in-hospital mortality [92].

It has been suggested that extracorporeal carbon dioxide removal (ECCO_2_R) can manage both hypoxemic and hypercapnic respiratory failure. ECCO_2_R uses a blood flow of around 0.5–1.5 L/min, allowing removal of low-flow CO_2_ and pH control while avoiding the invasiveness of ECMO. Some studies have suggested that ECCO_2_R is able to decrease MP and VILI and to maintain oxygenation [93,94]. High-flow VV-ECMO makes it more difficult to optimize oxygenation and CO_2_ removal given the higher flows adopted (2–4 L/min) [95]. Bein et al. [96] compared low V_T_ ventilation (3 mL/kg PBW) versus the ARDSNet strategy (6 mL/kg PBW) during ECCO_2_R and found that the use of very low V_T_ had a greater potential to further reduce VILI during ECCO_2_R. Morris et al. [97] concluded there was no significant difference in survival between the mechanical ventilation and the ECCO_2_R groups, suggesting that further investigations are needed to confirm whether ECCO_2_R is effective for ARDS. A recent systematic review of both RCTs and observational studies concluded that evidence is lacking to confirm beneficial effects of ECCO_2_R on outcome in ARDS, although positive insights about lung protective ventilation and VILI were found [98].

### 3.9. Fluid Management

The optimal fluid management in ARDS is still unknown. There are risks and benefits to liberal and conservative fluid management strategies.

FACTT was the defining trial testing the effect of a conservative fluid strategy in ARDS [99]. The trial adopted active diuresis, fluid bolus, vasopressor, and/or inotrope based on varying ranges of central venous pressure (CVP) and pulmonary artery occlusion pressure (PAOP). After a week, they found a significant difference in the cumulative fluid balance between the conservative and liberal de-resuscitation groups (−136 ± 491 mL versus 6992 ± 502 mL; *p* < 0.001). The daily cumulative fluid balance in the liberal group was similar to that in other contemporary ARDS trials (4 L and 6 L by day 4 in ARMA and ALVEOLI, respectively) and consistent with usual care at the time. They found no difference in 60-day mortality in these groups (25% with a conservative strategy versus 28% with a liberal strategy, *p* = 0.30). The conservative strategy group, however, had significantly more ventilator-free days (14.6 ± 0.5 versus 12.1 ± 0.5, *p* < 0.001) and ICU-free days compared with the liberal strategy group. Despite the aggressive conservative/de-resuscitation strategy, which targeted CVP lower than 4 mmHg and a PAOP lower than 8 mmHg, there was no increase in organ failure between the conservative and liberal arms of the study. Moreover, there were no significant differences in the percentage of patients receiving renal replacement therapy (10% in the conservative group versus 14% in the liberal group, *p* = 0.06) or the average number of days on renal support. Since this landmark study, practice in critical care has had a significant shift. These findings suggest that liberal fluid management may be more harmful in patients with ARDS by increasing pulmonary edema and prolonging mechanical ventilation days and ICU and hospital stay. Preventing fluid overload may lead to improved outcomes, and active de-resuscitation may mitigate the lung injury associated with excess intravenous fluids without compromising organ perfusion.

Different ARDS phenotypes may respond differently to fluid management. A recent secondary analysis of the FACTT trial suggests that hypoinflammatory and hyperinflammatory phenotypes could differ with regard to fluid responsiveness. In this study, sub-phenotype 1 was characterized by hypoinflammation and a higher proportion of white patients, whereas sub-phenotype 2 was characterized by hyperinflammation and hypotension. According to the sub-phenotypes, two different responses to distinct fluid strategies were found regarding outcome (*p* = 0.0039). In sub-phenotype 1, mortality was 26% with a liberal fluid strategy versus 18% with a conservative strategy. In sub-phenotype 2, mortality was 40% with a liberal fluid strategy versus 50% with a conservative fluid strategy [100]. Hence, it is key to determine the optimum volume status in each individual patient and to personalize the patient’s treatment according to the sub-phenotype.

## 4. Pharmacologic Therapies for ARDS

Over the years, several therapies have been tested in ARDS, targeting the pathophysiologic mechanism of ARDS and acting on the different phases of the disease. Several drugs have been tested to repair or limit alveolar epithelial damage, inflammation and immune response, edema and fibrosis, vascular remodeling, vascular permeability, and endothelial cell damage. Despite decades of investigating effective drug therapies for ARDS treatment, ARDS management remains mainly supportive with limited efficacy of the drugs explored in clinical practice and failed clinical trials [101,102,103,104,105,106,107,108,109,110,111,112,113,114,115,116,117,118,119,120,121,122,123,124,125,126,127,128,129,130,131,132,133,134,135,136,137,138,139,140,141,142,143,144,145,146,147,148,149,150,151,152,153,154,155]. An overview of mechanisms of action of the main drugs tested in ARDS research and their current use are provided in Table 1.

### 4.1. Neuromuscular Blocking Agents

Neuromuscular blocking agents (NMBAs) have been widely invest.igated in ARDS research. Their use is always associated with sedatives and analgesics to maintain paralysis under deep sedation and guarantee passive mechanical ventilation. However, in recent times, the ultimate goal of initial ARDS treatment is to provide early active breathing, thus reducing muscle wasting and improving oxygenation [139,140,141]. Despite the proven advantages of reducing active effort to improve oxygenation in patients with severe ARDS, meta-analyses have shown that there is still no consensus on the adoption of a strategy with early and continuous infusion of NMBAs or others comprising lighter sedation. When used for 48 h, NMBAs seem to improve oxygenation and reduce the risk of barotrauma in moderate to severe ARDS, without clear benefits on mortality, ventilator-free days, and duration of mechanical ventilation. The latest guidelines [102,103] concluded that NMBAs are considered in cases of early and severe ARDS with deep sedation, invasive mechanical ventilation, and the need for prone positioning within 48 h, but there is no evidence to support the routine and early use of NMBAs in ARDS. Further indications will come from three new trials investigating cysatracurium, which are currently recruiting (bolus versus continuous infusion, NCT05153525; NMBAs versus spontaneous breathing in patients under VV-ECMO, NCT04524585; and early NMBAs versus sedation alone, NCT04922814).

### 4.2. Corticosteroids

The role of corticosteroids in ARDS therapy is still controversial. It has been hypothesized that their potent anti-inflammatory effects have benefits in ARDS. In addition, substantial development during the COVID-19 pandemic confirmed potential benefits with corticosteroids for patients with severe COVID-19 ARDS. The evolution of testing steroids in ARDS research includes different steroid types, dosages, timing of initiation, and duration of therapy. In addition, several studies were performed at different time frames with regard to the advent of lung protective ventilation. Methylprednisolone has been tested early and late in the phases of the syndrome without providing clear benefits [105,106]. Rather than using methylprednisolone, in a recent trial, Villar et al. [142] tested dexamethasone, which improved survival. In the DEXA-ARDS trial, dexamethasone 20 mg once daily for 5 days followed by 10 mg once daily for 5 days increased ventilator-free days (between group difference. 4.8 days; 95% CI, 2.57–7.03, *p* < 0.0001) and reduced 60-day mortality significantly (21% vs. 46%). A recent meta-analysis from eight RCTs supported the use of corticosteroids for mortality benefits (relative risk, 0.71; 95% CI, 0.54–0.92) [104]. However, corticosteroids cannot be considered as standard of care in patients with ARDS, and the heterogeneity in responses among patients with ARDS is a possible reason for the uncertain response to this treatment.

### 4.3. Aspirin

Aspirin is an inhibitor of cyclooxygenase on platelets, which inhibits the receptor non-selectively, modulating inflammation [107]. Aspirin attenuates hyperoxia-induced ARDS by suppressing pulmonary inflammation via the nuclear factor (NF)-kβ signaling pathway [143]. Aspirin was tested as a preventive treatment in the evolution of ARDS in the LIPS-A trial, demonstrating neither reduced incidence of ARDS nor less ventilator days, length of stay in the ICU, and survival [108]. No studies investigating aspirin in patients with already established ARDS are available. However, in this trial, the number of patients who developed ARDS was low, as was the risk of evolution of the disease. There are currently two studies awaiting results: the STAR phase 2 trial (NCT02326350) and the ARENA trial (NCT01659307).

### 4.4. Interferons

Interferons are anti-inflammatory cytokines which act on the expression of cluster differentiation on the vascular endothelium. Two trials tested interferon-β and showed contrasting results. A phase 1 trial confirmed the efficacy of interferon-β with reduced 28-day mortality and improved gas exchange [109]. The INTEREST phase 3 trial [144] found no improvement in ventilator-free days and mortality when testing interferon-β for 6 days. However, the effect of interferon-β alone was not separated from those patients who received corticosteroids, creating a potential bias.

### 4.5. Vitamins

Vitamins D and C have been tested in ARDS. Vitamin D has an immunomodulator effect on innate and adaptive immunity, whereas vitamin C attenuates the expression of pro-inflammatory cytokines and inhibits nuclear factor kB. The administration of both vitamins showed negative results in ARDS patients. Vitamin D (VINDALOO trial) [110] did not reduce inflammation modulating biomarkers. In addition, in patients without ARDS (VIOLET trial) [145], no efficacy was confirmed on 90-day mortality. Regarding vitamin C, the CITRIS-ALI phase 2 trial found that a high-dose vitamin C infusion compared with placebo did not significantly limit organ failure at 96 h or improve inflammatory biomarkers [111]. Three trials testing vitamin C are currently enrolling (NCT04411160, NCT03780933, and NCT04404387).

### 4.6. Statins

Statins are currently tested in ARDS research because of their anti-inflammatory and immune properties. The HARP trial demonstrated that simvastatin could reduce inflammation and organ dysfunction [112]. However, McAuley et al. [146] did not find an improvement in ventilator-free days. According to personalized medicine, in a secondary analysis of the HARP-2 trial, the authors showed that ARDS sub-phenotypes responded differently to pharmacotherapies. Simvastatin improved survival in patients classified as hyperinflammatory sub-phenotype in comparison with hypoinflammatory [114]. These findings support the use of a personalized treatment approach. When re-analyzing the HARP-2 trial by sub-phenotypes, simvastatin was associated with greater survival in patients with higher inflammatory biomarkers [147]. In the SAILS trial, rosuvastatin did not improve outcome, and the study was stopped for futility [113]. In 2016, Dinglas et al. [148] evaluated patients from the SAILS trial over a 1-year follow-up. They found no significant difference in cumulative survival in the rosuvastatin versus placebo groups (58% versus 61%; *p* = 0.377). Survivors showed significant impairment of physical function and mental status without adjunctive beneficial effects when using rosuvastatin versus placebo on the SF-36 physical function test, the 6-min walk test, and mental health and other functional outcomes. Despite these negative results, ulinastatin demonstrated anti-inflammatory effects and antioxidant properties [149]. Two recent meta-analyses on statins confirmed benefits on mortality, duration of mechanical ventilation, length of ICU stay, organ failure, and the need for mechanical ventilation [115,116]. The NCT02895191 trial is currently ongoing and will provide more information about ulinastatin; the NCT03089957 trial comparing ulinastatin versus usual ICU care in patients at risk of developing ARDS is also ongoing. Unfortunately, there is still no clear recommendation for statins, because the trials used the same doses that showed efficacy in healthy volunteers but in critically ill patients [150].

### 4.7. N-Acetylcysteine

N-Acetylcysteine (NAC), an antioxidant, improves oxygenation [117] and reduces the need for ventilator support [151,152]. However, the potential advantage of NAC on hypoxemia was not always confirmed by clinical trials [118]. In addition, many years ago, Moradi et al. [153] found a significant positive effect on mortality. However, the trials testing NAC are old, and several things have changed over the years in the management of ARDS, thus limiting possible applicability in modern times. Recently, Taher et al. investigated the use of NAC in mild to moderate COVID-19 ARDS, finding no differences in 28-day mortality between NAC and placebo groups [119]. Modern trials, with modern designs, accounting for new ARDS definitions and patient’s sub-phenotypes are needed.

### 4.8. β-Agonists

β-Agonists are bronchodilator drugs and are proposed for the treatment of ARDS due to their anti-inflammatory properties and clearance of alveolar fluids. The main effect of β-agonists is improved oxygenation in patients with ARDS, but evidence of improved outcome is scarce [120,121]. The BALTI-2 trial compared salbutamol and placebo without finding any improvement in outcomes [154]. There are currently no trials ongoing. The identification of patients who may benefit from airway clearance could be a strategy to assess the real efficacy of β-agonists in new trials.

### 4.9. Sivelestat

Sivelestat, a neutrophil elastase inhibitor, acts by reducing pulmonary airway pressure and lung vascular permeability [122,123]. The main finding about sivelestat in patients with ARDS is the improvement in oxygenation and inflammation. However, in the STRIVE trial, a trend toward increased mortality was found, and the trial was stopped prematurely [124]. A meta-analysis confirmed potential benefits for gas exchange without evidence on mortality. This is probably because mortality was not the first aim of these trials, suggesting the need for updated and different targeted clinical studies [155].

### 4.10. Vasodilators

Inhaled nitric oxide (iNO), prostaglandins, and prostacyclins have been tested in patients with ARDS, providing different results. Guidelines suggest the use of iNO in the case of severe hypoxemia in severe ARDS despite the use of other rescue maneuvers (e.g., prone positioning), possibly as a bridge therapy to ECMO. Despite the confirmed benefits in improving oxygenation, there is no conclusive evidence on mortality outcome [125,126]. Similar to nitric oxide, prostaglandins and prostacyclins have vasodilatory properties. A recent trial confirmed there were no improvements in oxygenation or clear benefits on outcomes using treprostinil [127]. Aerosolized prostacyclin showed similar efficacy to iNO on pulmonary vasodilation and improvement of oxygenation [156]. Some trials have ended and are awaiting results: one investigating the effect of prostacyclin compared with saline on oxygenation and pulmonary artery vasodilation (NCT00314548) and another investigating the effect of iloprost versus placebo on mortality and oxygenation (NCT03111212); the effect of alprostadil is currently being tested in comparison with saline on both oxygenation and vascular thrombosis in patients with ARDS undergoing ECMO (NCT02895373).

### 4.11. Surfactants

Surfactants act by reducing alveolar surface tension, thus preventing alveolar collapse and limiting pulmonary edema. Surfactants also have anti-inflammatory and antimicrobial properties [150]. Findings on the impact of surfactants on mortality in ARDS have been conflicting over years, and a recent meta-analysis concluded no significant improvement of mortality and gas exchange [129]. In addition to the absence of effect, surfactants may cause hypoxemia and hypotension [128]. There are two phase 2 trials ongoing (NCT00215553 and NCT00682500-CARDS trial, both in children and adults) comparing the effect of calfactant versus placebo on 90-day mortality and duration of mechanical ventilation.

### 4.12. Solnatide

Solnatide or AP301, an inhaled peptide, reduces extravascular lung water and activates epithelial sodium channels, thus improving lung function. Promising efficacy has been shown in preclinical studies [130]. There is currently a phase 2 trial (NCT03567577) investigating the effect of inhaled solnatide at three different doses compared with placebo on all-cause mortality.

### 4.13. Dilmapimod

Dilmapimod, a p38 mitogen-activated protein kinase inhibitor, inhibits the release of pro-inflammatory cytokines. Christie et al. [131] demonstrated anti-inflammatory activity after continuous infusion; Yang et al. [132] tested the pharmacokinetic and pharmacodynamic properties of dilmapimod for the prevention of ARDS in patients at risk, without finding improvement. No other trials are currently ongoing.

### 4.14. Keratinocyte Growth Factor and Granulocyte-Macrophage Colony Stimulating Factor

Keratinocyte growth factor (KGF), a product of fibroblasts and T cells, inhibits apoptosis and has mitogenic effects. Two trials evaluated the effects of KFG but found no efficacy in reducing leukocyte infiltration or inflammation. In the KARE trial, KFG did not improve gas exchange and clinical outcome, and mortality was even higher than expected, suggesting potential harm [133,134].

Granulocyte-macrophage colony stimulating factor (GM-CSF) showed promising results in the preclinical setting, stimulating the maturation of alveolar epithelial cells [157]. However, no benefits were confirmed in clinical trials on ventilator-free days and mortality. There is currently a trial comparing GM-CSF with placebo that is investigating bronchoalveolar lavage fluids of patients with ARDS [135].

### 4.15. Nebulized Heparin

Nebulized heparin showed efficacy in dissolving thrombi and limiting the deposition of alveolar fibrin, which can be responsible for hypoxemia and altered alveolar capillary permeability [158]. A phase 3 trial testing the effects of unfractionated nebulized heparin 25,000 IU every 6 h to day 10 versus placebo found no improvement in daily physical activities but reduced progression of lung injury. A trial (NCT03465085) is currently testing the effect of nebulized heparin 10,000 IU every 4 h in comparison with streptokinase 250,000 IU every 4 h and placebo on gas exchange. No further trials are programmed.

### 4.16. Mesenchymal Stem Cells and Multipotent Progenitor Cells

Mesenchymal stem cells (MSCs) showed promising results in the preclinical setting thanks to their potential to modulate the immune response and reduce lung injury [136]. A phase 1 trial was conducted demonstrating the safety profile of MSCs [137]. However, 28-day mortality did not differ between the groups (30% in the MSC group versus 15% in the placebo group; odds ratio, 2.4; 95% CI, 0.5–15.1). After adjusting for APACHE III scores, the hazard ratio for mortality was 1.43 (95% CI, 0.40–5.12, *p* = 0.58). One dose of intravenous MSCs was safe in patients with moderate to severe ARDS. Larger trials are needed to assess efficacy, and the viability of MSCs must be improved, but no clear benefits of MSCs have been confirmed to date. Another trial on the safety of MSCs and clinical improvement has terminated (NCT02804945).

A recent phase 1/2 trial from Bellingan et al. (MUST-ARDS) [138] evaluated the safety and tolerability of intravenous multipotent adult progenitor cells in patients with moderate to severe ARDS. In the phase 2 trial, the administration of 900 million cells was compared with placebo within 96 h of an ARDS diagnosis; 28-day mortality was 25% for the cell group versus 45% for placebo recipients. In addition, 28-day liberation from the ICU and ventilator-free days were better in the cell group versus the placebo group.

## 5. Why Do Pharmacotherapies Fail in ARDS? The Importance of Personalized Medicine

**Figure 2:**
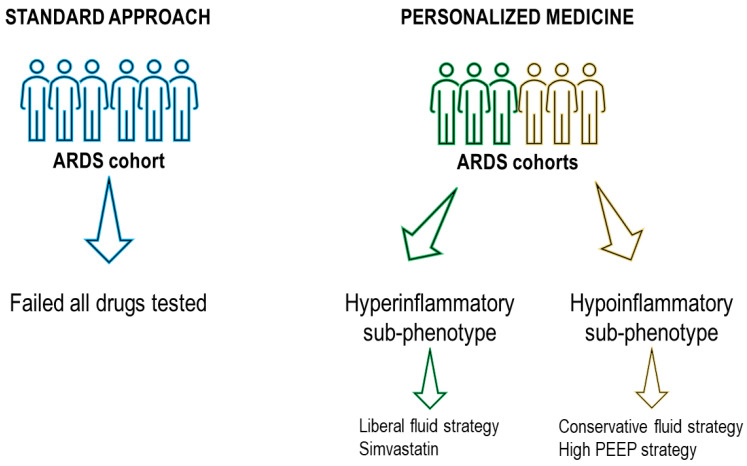
Personalized medicine approach versus standard approach. ARDS, acute respiratory distress syndrome; PEEP, positive end-expiratory pressure. Applying personalized medicine using latent class analysis, two sub-phenotypes are identified, which positively respond to some pharmacotherapies and supportive treatments that previously failed to demonstrate benefits in a broader and inhomogeneous ARDS cohort.

Supportive treatments have shown some positive results in the treatment of ARDS over the years. However, pharmacotherapies have not presented similar benefits [150], which may be attributed to the heterogeneity of the disease. Current knowledge about the design of clinical trials for ARDS suggest that recognition of the disease was inaccurate and untimely in old trials: investigated patients at risk of ARDS versus patients with established ARDS did not account for etiology in selecting the drug to test, did not focus on patient variability (different presentations, distinct phenotypes and genotypes) or heterogeneity in treatment approaches (standard of care might be different across countries), and did not apply novel trial designs (i.e., Bayesian analysis, platform trials, adaptive trials) [150]. When accounting for the heterogeneity of ARDS, some positive results on testing drugs that previously failed to demonstrated efficacy were achieved. A group of researchers re-analyzed five randomized controlled trials in adult patients, one in pediatrics, and two observational studies in which the original results were negative; using a new approach based on a machine learning model, the efficacy of failed therapies was found by sub-phenotyping ARDS [159]. Thanks to this approach, researchers found two sub-phenotypes: hyper- and hypoinflammatory. A secondary analysis by Liu et al. [160] of the ALVEOLI trial showed that patients with phenotype I (fewer abnormal laboratory values and less organ failure) had fewer ventilator-free days and ICU-free days with a high PEEP strategy. However, in the LIVE study, Constantin et al. [37] did not confirm that a personalized mechanical ventilator strategy tailored to lung morphology, as determined by computed tomography, is associated with better survival rates. In a re-analysis of the HARP-2 trial testing simvastatin, Calfee et al. [114] found higher survival using simvastatin in the hyperinflammatory phenotype. In a re-analysis of the FACTT trial [100], a conservative or liberal strategy with fluids produced contrasting results according to the phenotype. However, when testing rosuvastatin according to the phenotype, no differences were found. When analyzing the FACTT cohort, the fluid-conservative strategy was associated with improved mortality in phenotype II (higher white blood cell count, heart rate, RR, lower systolic blood pressure, and younger age) but had the opposite effect in phenotype III (older age, increased serum creatinine, blood urea nitrogen levels, and lower serum bicarbonate levels) [160]. However, when re-analyzing the SAILS study with a latent class analysis approach [161], no beneficial effects were found within sub-phenotypes. This raises the question whether the method of sub-phenotyping ARDS should be improved (Figure 2).

By carefully examining candidates for inclusion in clinical trials and looking into ARDS risk factors, phenotypes, and biological pathways as part of the study design, it may be possible to increase the effectiveness of the therapies under investigation while hopefully avoiding the premature discard of potentially helpful treatments. The classification of critically ill patients with ARDS according to subtype, endotype, phenotype, and biomarkers may therefore be very beneficial. For patients with advanced cancer, this approach has been effective [162]. The easy viability of this strategy, however, may be constrained by the fact that critically ill patients are among the population hardest to stratify due to coexisting underlying processes and disorders. ARDS clinical trial design methodologies need to be reconsidered. Clinical trial implementation in ARDS may benefit from the following strategies: (1) identification of the appropriate patient subset; (2) clinical trial enrichment strategies (e.g., “practical enrichment” such as choosing patients who will undoubtedly adhere to the treatment, are not taking any other medications similar to those in question, and are unlikely to die from other diseases; “prognostic enrichment” such as identifying high-risk patients; and “predictive enrichment” such as choosing patients who are likely to respond to treatment); (3) identifying biomarkers, risk factors, phenotypes, and endotypes to use in patient selection; (4) including biomarkers in the clinical study’s goals; (5) using an adaptive randomization strategy; (6) developing machine-learning models with new intriguing statistical analysis; (7) using preclinical models which reproduce ARDS more accurately; and (8) choosing the right patients [101,150].

## 6. Future Directions

Several questions remain unresolved regarding supportive therapies, including the use of spontaneous breathing, limiting V_T_ in patients who are breathing spontaneously, setting V_T_ according to lung volume, setting PEEP according to perfusion, minimization of asynchronies to improve outcome, association of mechanical power with outcome, higher PEEP not inferior to prone positioning, APRV to be used over conventional mechanical ventilation, ECCO_2_R to be used to facilitate ultra-protective lung ventilation or facilitate spontaneous breathing, or expand the use VV-ECMO beyond cases of very severe ARDS, and so on [163]. All these points will certainly be investigated in the next future.

Regarding pharmacologic therapies, latent class analysis and the identification of biological phenotypes seem promising for the future of ARDS to find effective therapies. Another new frontier is the omics approach. However, it is still difficult to apply at the bedside and needs further investigation [164].

Personalized medicine is difficult to implement in a real-world setting. Several strategies should be considered in the design of clinical trials to test the efficacy of a personalized approach. Some questions remain: (1) how to best select the patients; (2) how fast readout parameters can be easily and quickly measured; (3) how to allocate patients to receive one therapy instead of another; (4) how to access fast and feasible biomarker kits; and (4) how to implement administrative care.

## 7. Conclusions

Although more than 50 years have passed since the first definition of ARDS and several trials have been performed, we do not yet have an effective pharmacologic therapy. The future of ARDS in the era of precision medicine strives toward identifying treatable traits, thus looking for ARDS etiology, physiology, and sub-phenotypes. Biological markers and multi-omics approaches are potentially profitable strategies that need to be further investigated.

## Figures and Tables

**Table 1:** Mechanisms of action and current status of the main drugs tested in ARDS.

Drug.	Rationale for Using in ARDS	Current Status
NMBAs	NMBAs paralyze skeletal muscles by blocking the transmission of nerve impulses at the myoneural junction [102,103].	Considered in cases of early and severe ARDS with deep sedation, invasive mechanical ventilation, and the need for prone positioning within 48 h. There is no evidence to support NMBAs routine and early use [102,103].
Corticosteroids	Anti-inflammatory protein expression is regulated in the nucleus by the activated glucocorticoid receptor–glucocorticoid complex, thus reducing inflammation [104].	Not approved as a medical treatment guideline with no clear benefits in outcome. According to current research [104,105,106], there could be a significant patient benefit and the risk of adverse events is thought to be low. However, clinical trials, the majority of which were carried out before the advent of lung protective ventilation strategies, provided controversial results. Corticosteroids may be beneficial for certain steroid responsive illnesses that resemble ARDS.
Aspirin	Aspirin acts on platelet aggregation via inhibition of platelet thromboxane A2-synthesis. In ARDS, aspirin reduces pulmonary neutrophil infiltration as well as alveolar inflammation and injury [107].	Not approved. No clear benefits in outcome [107,108].
Interferons	Interferons are anti-inflammatory cytokines In ARDS, they facilitate clearance of bacteria, neutrophil apoptosis and efferocytosis, and promote lung repair [109].	Not approved. No clear benefits in outcome [109].
Vitamins	Vitamin D has an immunomodulator effect on innate and adaptive immunity [110], whereas vitamin C attenuates the expression of pro-inflammatory cytokines and inhibits nuclear factor kB [111].	Not approved. No clear benefits in outcome [110,111].
Statins	Statins act via inhibition of hydroxymethylglutaryl-coenzyme A reductase and also have many other pleiotropic effects, such as anti-inflammatory and anti-proliferative effects on lung inflammation [112].	Not approved. No clear benefits in outcome [113] Statins may probably have different effects according to patient’s sub-phenotype [114,115,116].
N-acetylcysteine	N-acetylcysteine acts as an antioxidant [117].	Not approved. No clear benefits in outcome [118,119].
β-Agonists	β-Agonists reduce bronchospasm, airway resistance, and inflammation as well as improve alveolar fluid clearance and stimulate alveolar epithelial and endothelial repair, thus benefiting pulmonary mechanics [120,121].	Not approved. No clear benefits in outcome [120,121].
Sivelestat	Sivelestat is an inhibitor of human neutrophil elastase. In ARDS, it improves oxygenation and reduces inflammation [122,123].	Not approved. No clear benefits in outcome [124].
Vasodilators	Nitric oxide activates soluble guanylyl cyclase (sGC) to produce cyclic guanosine monophosphate (cGMP). It improves oxygenation by increasing perfusion to well-ventilated lung regions as well as presents anti-inflammatory effects [125,126]. Prostaglandins have vasodilatory properties [127].	Not approved. No clear benefits in outcome [125,126,127].
Surfactants	Surfactants act by reducing alveolar surface tension, thus preventing alveolar collapse and limiting pulmonary edema. Surfactants also have anti-inflammatory and antimicrobial properties [128].	Not approved. No clear benefits in outcome [128,129].
Solnatide	*Solnatide* is a synthetic peptide mimicking the lectin-like domain of tumor necrosis factor. In ARDS, it reduces extravascular lung water (edema) and activates epithelial sodium channels, increases occludin expression, thus improving lung function [130].	Not approved. No clear benefits in outcome [130].
Dilmapimod	Dilmapimod is a p38 mitogen activated protein kinase. It reduces the levels of proinflammatory cytokines and chemokines as well as cell infiltration to inflammation sites [131].	Not approved. No clear benefits in outcome [132].
KGF and GM-CSF	KGF is a mitogen for specific different types of epithelial cells. In ARDS, KFG inhibits apoptosis and has mitogenic effects. GM-CSF stimulates maturation of alveolar epithelial cells [133,134].	Not approved. No clear benefits in outcome [133,134,135].
Nebulized heparin	In ARDS, nebulized heparin improves oxygenation and reduces lung edema [155].	Not approved. No clear benefits in outcome [155].
MSCs	MSCs modulate the immune response and reduce lung injury [136].	Not approved. No clear benefits in outcome [136,137,138].

Table Legend: ARDS = acute respiratory distress syndrome; NMBAs = neuromuscular blocking agents; KGF = keratinocyte growth factor; GM-CSF = granulocyte-macrophage colony stimulating factor; MSC = mesenchymal stromal cells.

## Data Availability

Not applicable.
